# Influence of Dietary Alpha-Tocopheryl Acetate (Vitamin E) and Animal Fat on the Chemical Composition, Fatty Acid Profile, Lipid Stability and Sensory Traits of Fresh and Stored Hamburgers from Rabbit Meat †

**DOI:** 10.3390/ani15121804

**Published:** 2025-06-19

**Authors:** Bianca Palumbo, Maria Elena Cossu, Antonella Dalle Zotte

**Affiliations:** 1 Department of Animal Medicine, Production and Health, Agripolis, University of Padova, 35020 Padova, Italy; biancafederica.palumbo@phd.unipd.it; 2 Department of Animal Production, Faculty of Agronomy, University of Buenos Aires, Buenos Aires C1053ABH, Argentina

**Keywords:** rabbit nutrition, antioxidants, oxidative stability, refrigerated storage, flavor enhancement, nutritional enrichment, shelf life

## Abstract

Improving the proportion of polyunsaturated fatty acids (PUFAs) in meat enhances its nutritional value; however, because PUFAs are prone to oxidation, incorporating antioxidants into animal diets may be an effective way to limit lipid degradation. This study investigated the effects of dietary fat content, vitamin E supplementation, and storage time on the oxidative stability, physico-chemical, sensory, and technological traits of rabbit hamburgers. Seventy-two rabbits (aged 40–78 days) were assigned to four groups: F0-E0 (0% pork fat, 0 mg/kg vitamin E), F0-E200 (0% fat, 200 mg/kg vitamin E), F2-E0 (2% fat, 0 mg/kg vitamin E), and F2-E200 (2% fat, 200 mg/kg vitamin E). After slaughter and carcass deboning, fat-free meat was processed into hamburgers. Three hamburgers per rabbit were analyzed immediately (T0) and three after 7 days of storage at 4 °C (T7). Vitamin E supplementation significantly reduced TBARS values (*p* < 0.001) and improved meat appearance and texture. Fat-enriched diets modified the fatty acid profile, increasing unsaturated and n-3 fatty acids, and enhanced meat flavor. Vitamin E supplementation improved the nutritional quality of the lipids by increasing PUFAs and n-3 FA levels. Storage time significantly affected quality traits, with T7 hamburgers showing higher TBARS values (*p* < 0.001) and a greater UFA percentage.

## Simple Summary

Improving the healthiness of meat is an important goal for both consumers and producers. One way to do this is by increasing the amount of healthy fats. However, these fats can spoil easily, especially during storage. This study looked at whether adding vitamin E, a natural antioxidant, to rabbit feed could help protect these fats and improve meat quality. Rabbits were fed different diets—some with added fat, some with vitamin E, and some with both. The meat was processed into hamburgers and tested immediately and after one week in the fridge. The results showed that vitamin E helped reduce fat spoilage and improved the look and texture of the meat. Diets enriched with fat increased the levels of beneficial fats and enhanced the flavor of the meat. However, storage over time led to a decline in quality. This research demonstrates that combining healthy fats with antioxidants in animal diets can lead to higher-quality, more nutritious meat products. These results offer valuable insights for farmers and food producers aiming to improve meat quality while reducing waste caused by fat degradation.

## 1. Introduction

Rabbit meat is widely recognized for its high nutritional quality, particularly its favorable lipid profile, which features a high ratio of polyunsaturated fatty acids (PUFAs) to saturated fatty acids (SFAs) [1]. PUFAs, encompassing omega-3 and omega-6 fatty acids (FAs), are essential components of the human diet, playing pivotal roles in various physiological functions and offering numerous health benefits [2,3]. This explains the interest in obtaining meats rich in these kinds of fatty acids, able to improve the nutritional profile of meat [4,5,6]. Additionally, to meet the energy requirements and improve feed efficiency in rabbits, particularly in commercial hybrids, dietary fat can be incorporated. This practice increases body fat content, enhances live weight, and alters certain carcass characteristics. Both animal and vegetable fats can be provided to rabbits; however, the inclusion of animal fat facilitates the pelleting process of the feed [7], and since it contains a higher proportion of saturated fatty acids, it increases the saturated fat content in the meat, reducing its vulnerability to oxidative degradation [8]. This complex process involves the degradation of lipids, leading to the formation of off-flavors, color changes, and potentially harmful compounds, thereby reducing the storage time of meat. The susceptibility to oxidation is particularly high in phospholipids, which are rich in PUFAs and constitute a significant portion of muscle cell membranes. Intramuscular lipids are also subject to oxidative processes, catalyzed by endogenous pro-oxidants such as myoglobin, cytochromes, and non-heme iron present in the meat [9,10]. Understanding these mechanisms is crucial for developing strategies to mitigate lipid oxidation in meat products. Approaches such as dietary supplementation with antioxidants, including vitamin E, have been explored to enhance the oxidative stability of meat [11,12]. Vitamin E, primarily in the form of α-tocopheryl acetate, functions as a potent lipid-soluble antioxidant that protects muscle tissues from oxidative damage during storage [10]. In broiler chickens, dietary inclusion of 200 mg/kg α-tocopheryl acetate significantly decreased thiobarbituric acid reactive substances (TBARSs) during storage [13]. A similar effect was observed with dietary inclusion of 500 mg/kg of vitamin E in a pig diet [14]. Furthermore, studies on rabbits have shown that vitamin E is an efficient antioxidant in cell membranes and reduced lipid oxidation of meat [15,16,17,18]. Previous studies have shown that supra-nutritional supplementation with α-tocopheryl acetate enhances the oxidative stability of both raw and cooked meat, as indicated by reduced lipid peroxidation and greater retention of n-3 PUFA [19]. In contrast, relatively few investigations have explored whether similar dietary vitamin E enrichment can safeguard the quality attributes of rabbit meat once it has been processed into value-added products. To date, only a limited number of reports have addressed this question, underscoring the need for further research on the efficacy of α tocopherol in maintaining the technological, sensory, and nutritional properties of processed rabbit meat [20].

Based on the foregoing, an experiment was conducted to investigate how rabbit diets differing in pork fat level and α-tocopheryl acetate supplementation influence growth performance, carcass traits, and meat quality, and to determine whether dietary vitamin E enhances the oxidative stability of muscle and hamburger meat. The study also examined how storage duration affects the physico-chemical, sensory, and technological properties of the hamburgers. Results on growth performance, carcass yield, and overall meat quality have been reported previously [21]. The present paper is an extended version of [22] and it analyses selected quality attributes of raw and cooked rabbit hamburgers.

## 2. Materials and Methods

### 2.1. Animals and Diets

A detailed description of the experimental site, feeding, animals, and their management is provided by a previous study [21]. All rabbits were treated in accordance with the European Commission Directive 86/609/EEC on the protection of animals used for experimental and other scientific purposes. The present study was conducted as a continuation of the aforementioned work [21], with a focus on both postmortem and in vivo measurements. All procedures were performed as part of routine farm management and did not involve any invasive or stressful interventions. As such, ethical approval from the relevant committee was not required.

A total of seventy-two weaned (at 35 d of age) hybrid rabbits (Grimaud Frères) of both sexes were individually housed in cages (28 × 41 × 28 cm) under controlled environmental condition. At 49 days of age, the rabbits were randomly allocated into four groups, each consisting of 18 replicates, and were fed ad libitum with experimental diets varying in pork fat and vitamin E (α-tocopheryl acetate) content. The diets included the following: a control diet with no added fat or vitamin E (F0-E0); a fat-free diet supplemented with 200 mg/kg of vitamin E (F0-E200); a diet containing 2% pork fat without vitamin E supplementation (F2-E0); and a diet containing 2% pork fat and 200 mg/kg of vitamin E (F2-E200). The ingredients and chemical composition of the diets are described in detail in Tables S1 and S2, respectively, whereas the percentage of total fatty acid methyl esters (FAMEs) of the diets is presented in Table 1.

### 2.2. Meat Sampling and Physicochemical Analyses

At 78 days of age, 15 rabbits per group were weighed, electrically stunned (90 V × 2 s), and slaughtered at an EU-licensed abattoir. The slaughtering and carcass dissection procedures followed the World Rabbit Science Association (WRSA) recommendations described by [23]. From the reference carcasses (RCs) (obtained from chilled carcasses after removing the head, thoracic cage organs, liver, and kidneys), perirenal fat, scapular fat, and other dissectible fat were dissected and weighed, and their ratio to the RC were calculated as required. Meat from each RC was ground using a meat grinder (ABO, Milan, Italy) twice and used to prepare 6 hamburgers per rabbit, each weighing approximately 50 g, which were then subjected to physical and sensory analyses. Three hamburgers per rabbit were immediately analyzed (T1) for color and pH, while the other three were placed in polystyrene trays, over-wrapped with a traditional polyvinyl chloride film, and analyzed for the same parameters after 7 days of storage (T7) at 5 ± 2 °C. For sensory analyses, one fresh hamburger and one stored hamburger per rabbit were used. At 24 h postmortem, the ultimate pH (pHu) was determined in situ with a portable pH meter (HI98161, Hanna, Padua, Italy) equipped with a glass electrode (3 mm-diameter conic tip) suitable for meat penetration. Instrumental meat color expressed as L* (lightness), a* (redness), b* (yellowness), and C* (Chroma), according to the CIElab system [24], was measured with a Minolta CR300 chromameter (Minolta, Osaka, Japan) on the hamburger surface; the Illuminant was D65, and an incidence angle of 0 was used. The values corresponded to the average of three measurements per sample.

The proximate composition of hamburgers was performed according to the Association of Official Analytical Chemists [25] to determine the dry matter (method No. 934.01), crude protein (method No. 2001.11), and ash (method No. 967.05) contents. The ether extract was determined after acid hydrolysis (EC 1998) [26].

In order to determine the cooking loss, the hamburgers were individually packed in sealed bags and cooked in a water bath (M428-BM, MPM Instruments, Bernareggio, Italy) at 80 °C for 50 min [27]. The FAs in the meat were extracted according to [28] and determined as methyl esters with a GC (HRGC 5300 Megaseries, Carlo Erba, Milan, Italy) using a Supelco Omegawax 250 capillary column (30 m, 0.25 mm ID, 0.25 μm film thickness). The PCL/PCE index was calculated [29,30].

Lipid oxidation of fresh and stored hamburgers was determined through the evaluation of the thiobarbituric acid-reactive substances (TBARSs). The absorbance of samples was read at 532 nm using a spectrophotometer (Hitachi U-200, Hitachi, Mannheim, Germany). The TBARS values were calculated from a standard calibration curve of 1, 1, 3, 3-tetraethoxypropane, and the values were expressed as mg of malondialdehyde (MDA)/kg of sample [31]. The vitamin E level of one hamburger per treatment was analyzed by the company “Istituto delle Vitamine SpA” (DSM group), Milano, Italy; the sample was hydrolyzed with ethanolic KOH, and vitamin A was extracted with petroleum ether. After solvent evaporation, the residue was dissolved in methanol and diluted if necessary. Vitamin A was quantified by RP-HPLC with UV or fluorescence detection, using conditions that do not separate all-trans and cis isomers. To obtain a representative sample, hamburgers from each treatment were pooled for both the T0 and T7 time points.

### 2.3. Sensory Analysis

Color and appearance were evaluated in raw hamburgers, whereas appearance, flavor, and texture (tenderness and juiciness) were assessed in cooked hamburgers. Sensory analysis was carried out by a trained panel of 10 individuals, specifically trained in both the product (rabbit meat) and the methods used for sensory evaluation. The panel assessed raw and cooked meat samples sequentially using a ranking classification system [32], under standardized conditions [27]. The panelists evaluated the samples over two sessions, with each panelist tasting four samples per session. In the first session, they ranked the fresh hamburgers (T1) using a scale from 1 to 4 (from least to most) based on the color and appearance of the raw meat, as well as the appearance, flavor, and texture of the cooked hamburger. In the second session, the same evaluation was conducted for the stored hamburgers (T7).

### 2.4. Statistical Analysis

All variables were first tested for normality using the PROC UNIVARIATE procedure of SAS v 9.1.3 software [33] (SAS Institute, Cary, NC, USA, 2008). Normal distribution was evaluated based on the Shapiro–Wilk test. Homogeneity of variances was assessed using Levene’s test. Since the assumptions of normality and homoscedasticity were met, data were analyzed by analysis of variance (ANOVA) using the PROC GLM procedure of SAS software according to the following model:

Y_ijkl_ = μ + α_i_ + β_j_ + γ_k_ + (βγ)_jk_ + ε_ijkl_

where

Y_ijkl_ = observed value of the dependent variable;
μ = overall mean;
α_i_ = fixed effect of storage time (i-th level);
β_j_ = fixed effect of vitamin E level (j-th level);
γ_k_ = fixed effect of fat level (k-th level);
(βγ)_jk_ = interaction effect between vitamin E and fat level;
ε_ijkl_ = random residual error, assumed to be normally distributed with mean 0 and constant variance σ^2^.

Differences among least squares means (LS-means) were evaluated using the Bonferroni-adjusted *t*-test. Statistical significance was accepted at *p* ≤ 0.05.

## 3. Results and Discussion

### 3.1. α-Tocopheryl Acetate Level

**Figure 1:**
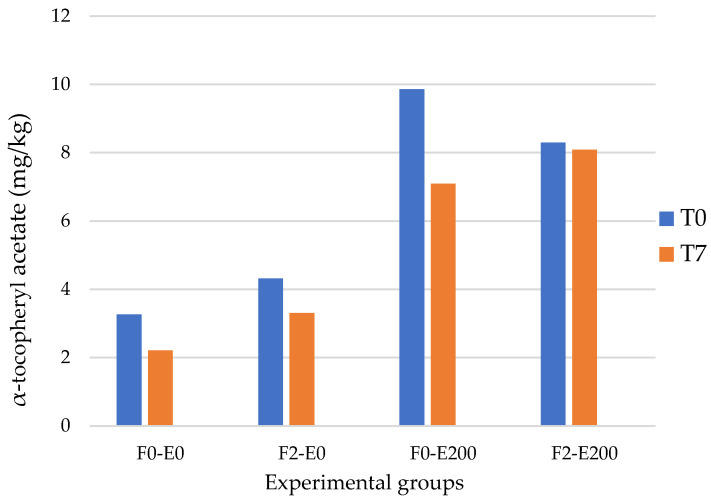
Content of α-tocopheryl acetate (mg/kg) of fresh (T0) and stored (T7: 7 days) rabbit hamburgers.

In both fresh and stored hamburgers, vitamin E concentration exceeded that reported for the longissimus dorsi (LD) muscle by [21], a difference attributable to the higher fat content of the hamburgers (averaging 8.34 mg kg^−1^ in raw patties versus 3.49 mg kg^−1^ in LD. Among rabbit hamburgers, the fresh ones had a higher concentration of vitamin E. Storage time, indeed, had an effect on the vitamin E content of the hamburgers (Figure 1). The vitamin E content in T7 patties was, on average, 21% lower than that of T1 samples, likely due to the natural role of vitamin E in antioxidant processes. In contrast to the present findings, [34] reported that vitamin E levels in α-tocopherol-fortified chicken sausages remained relatively stable during 10 days of refrigerated storage at 4 °C, with no significant losses observed. Conversely, a longer storage period resulted in a 58% reduction in vitamin E content in beef burgers and a 26% reduction in chicken frankfurters, as reported by [35]. The concentration of vitamin E in meat products generally declines over time due to oxidative processes, processing methods, and storage conditions. Vitamin E is a lipid-soluble antioxidant that is particularly sensitive to various environmental and processing factors. Its stability can be significantly influenced by storage temperature, duration, oxygen exposure, and the type of packaging used. In the present study, the reduction in vitamin E content observed in rabbit hamburgers at the T7 time point is likely the result of a combination of these factors (Figure 1). Among them, the presence of oxygen within the packaging plays a particularly critical role, as it promotes oxidative reactions that lead to the degradation of tocopherols.

### 3.2. Physico-Chemical Traits of Hamburger

Table 2 and Table 3 show the effects of vitamin E supplementation (E200), animal fat inclusion (F2), and storage duration (T, in days) on the physico-chemical characteristics of raw and cooked hamburgers, respectively. Although the inclusion of α-tocopheryl acetate had no significant effect on the parameters evaluated, storage time had a notable impact on the color of raw hamburgers. In contrast, no significant changes in pH were observed over the storage period. In particular, prolonged storage resulted in a reduction in lightness (L*) (*p* < 0.001), redness (a*) (*p* < 0.001), yellowness (b*) (*p* < 0.001), and color intensity (C*) (*p* < 0.001) of raw hamburgers. This change is likely due to pigment oxidation, which contributes to the development of a paler, more greyish appearance—characteristic of rabbit meat (Table 2). During the first 24 h postmortem, studies have reported an increase in L*, a*, and b* values [36,37]. However, in agreement with the present findings, [38] found that with extended storage (2, 8, and 12 days of refrigeration), the pH of rabbit meat remained stable, while L*, b*, and chroma (C*) values showed a decreasing trend over time. During storage, myoglobin can oxidize, leading to the formation of metmyoglobin, which imparts a brownish hue to the meat. This oxidation process is influenced by factors such as oxygen exposure, temperature, and pH levels. Additionally, microbial activity can contribute to discoloration by producing metabolites that affect meat pigments [36,39].

In cooked hamburgers, dietary inclusion of α-tocopheryl acetate did not significantly influence any of the evaluated parameters. However, storage duration had a marked effect on color. Specifically, an increase in lightness (L*) was observed in hamburgers from rabbits fed the F2-E0 and F2-E200 diets (*p* < 0.01 and *p* = 0.05, respectively). This effect may be attributed to the higher fat content in these groups, which could have influenced the water-holding capacity, surface light reflectance, or fat distribution following storage and cooking. The observation that dietary fat level affected lightness during storage, while vitamin E supplementation had no significant effect after cooking, suggests that dietary antioxidants are more effective in preserving the quality of raw meat. In contrast, post-cooking color characteristics appear to be more influenced by storage conditions and dietary fat content than by the meat’s initial antioxidant status. Similar to the trend observed in raw hamburgers, redness (a*), yellowness (b*), and overall color intensity (C*) decreased over the storage period for all the treatment groups. No significant effects of storage or dietary treatment were observed on cooking loss values.

The proximate composition of the hamburgers is reported in Table 4. Results are consistent with those reported in previous studies [1,40,41]. No significant effects of dietary treatment or storage time were observed, except for the protein content, which increased with storage duration in the F0-E200 and F2-E200 groups (*p* < 0.05). Vitamin E helps mitigate oxidative stress and modulate inflammatory responses, thereby indirectly preserving protein integrity during storage [42]. By scavenging reactive oxygen species (ROS), vitamin E may reduce protein oxidation, contributing to the observed increase in or preservation of protein content in vitamin-E-supplemented groups.

### 3.3. Sensory Analysis of Hamburger

Table 5 presents the sensory evaluation of rabbit hamburgers. Patties obtained from animals whose diets were enriched with vitamin E received significantly higher appearance scores than those from the non-supplemented groups, both in fresh meat (2.63 vs. 2.37; *p* < 0.05) and after seven days of chilled storage (2.73 vs. 2.28; *p* < 0.05). Ground meat is inherently prone to oxidative and color deterioration because of its large surface area, but vitamin E is preferentially deposited in muscle and adipose tissues, where it enhances lipid and pigment stability [43]. The sensory benefits of dietary vitamin E extended to the cooked product as well: panelists rated the supplemented hamburgers noticeably more tender and juicier than the controls (2.74 vs. 2.27; *p* < 0.05). This result, attributed to the positive effect of vitamin E on the water-holding capacity, appears particularly interesting from a technological perspective, considering the growing trend in marketing rabbit meat in the form of processed or precooked products. Dietary fat enrichment produced a general improvement of the sensory characteristics of hamburgers, but it was significant only in hamburgers stored under refrigeration for 7 days (Table 5). In particular, fat supplementation significantly increased the appearance score of raw hamburgers (2.72 vs. 2.28; *p* < 0.05) and the texture of cooked ones (2.80 vs. 2.20; *p* < 0.01). The favorable effect on the texture could depend on the increased lipid infiltration of meat derived from animals fed diets added with fat; as is well known, stimulating salivation increases the sensation of juiciness perceived by the consumer [44].

The combined supplementation of vitamin E and pork fat significantly lowered the color score of fresh hamburgers (*p* < 0.05), whereas their appearance received intermediate ratings. By day 7 of storage, however, hamburgers enriched with both vitamin E and pork fat showed a markedly enhanced flavor (*p* < 0.01).

### 3.4. Fatty Acid Profile of Hamburger

Table 6, Table 7, Table 8 and Table 9 report the FA profile (% total FAMEs) of raw hamburgers. No significant effect of dietary treatment was observed on the overall proportion of SFAs, except for C10:0, C15:0, and C18:0 (Table 6). The inclusion of pork fat in the diet reduced the content of capric acid (C10:0) at T7 (*p* < 0.05) but increased the content of stearic acid (C18:0) at T0 (*p* < 0.01). These findings are consistent with the known fatty acid composition of pork fat, which is rich in long-chain SFAs such as stearic acid and contains relatively low amounts of short-chain SFAs like capric acid [45]. Conversely, dietary supplementation with vitamin E resulted in a significant reduction in pentadecanoic acid (C15:0) content at T7 (*p* < 0.05).

Regarding monounsaturated fatty acids (MUFAs), the addition of pork fat decreased the content of palmitoleic acid (C16:1) at both T0 and T7 (*p* < 0.05) (Table 7). In contrast, it significantly increased the content of oleic acid (C18:1 n-9) of the hamburgers at both time points (*p* < 0.001). Oleic acid is a predominant MUFA in pork fat, and its elevated levels in the meat reflect the dietary inclusion of this lipid source. Ref. [46] reported that although oleic acid is partly synthesized endogenously, its concentration in meat is closely linked to the type and amount of dietary fat. Specifically, diets supplemented with beef tallow or pork lard, both rich in C18:1, resulted in increased deposition of this fatty acid in muscle tissues. Similar findings were documented by [47], who observed elevated C18:1 levels in meat following the use of olive oil, another oleic-acid-rich lipid source.

In contrast, the dietary inclusion of vitamin E led to a reduction in oleic acid content at T0 (*p* < 0.05) and in gondoic acid (C20:1 n-9) at the same time point (*p* < 0.05). The reduction in these MUFAs due to vitamin E supplementation may reflect a dilution effect or altered incorporation efficiency of MUFAs in the presence of high antioxidant levels [48,49]. No significant effect of the dietary treatments was observed on the proportion of PUFAs. In agreement with previous studies [50], linoleic acid (C18:2 n-6) was the most abundant PUFA, with an average proportion of 20.7% (Table 8).

Storage time had a significant effect on the fatty acid composition of rabbit hamburgers. In general, storage led to an increase in the content of most examined fatty acids, although some exceptions were noted. Among SFAs, the proportion of palmitic acid (C16:0), the most abundant, decreased over time (*p* < 0.001) (Table 6), consistent with the findings of [50]. In contrast, [49] reported no change in C16:0 levels before and after storage. The content of oleic acid (C18:1 n-9), which was approximately 25.5% at T0, aligned with values reported in previous studies [49]. However, consistent with the findings of [50], oleic acid levels in the present study declined after 7 days of storage in the F0-E0 and F2-E0 groups (*p* < 0.001 and *p* < 0.01, respectively).

Among PUFAs, a reduction in dihomo-γ-linolenic acid (C20:3 n-6) in groups F0-E0 and F2-E0 (*p* < 0.05 and *p* < 0.01, respectively) was noted with the storage of the hamburgers along with docosadienoic acid (C22:2 n-6; *p* < 0.001) in group F2E0, while the other PUFAs increased accordingly to [50] (Table 8). The increase in certain fatty acids during the storage of rabbit meat is primarily due to enzymatic hydrolysis of complex lipids, influenced by storage conditions and packaging methods. Triglycerides and phospholipids have varying susceptibilities to hydrolysis. Phospholipids, which are rich in PUFAs, are more prone to enzymatic degradation, leading to a relative increase in free PUFAs during storage [51]. The supplementation of α-tocopheryl acetate in the rabbit diet resulted in an increased proportion of PUFAs at T7 (*p* < 0.05), along with a significant rise in n-6 FA at the same time point (*p* < 0.05) (Table 9). A comparable trend was also observed in chickens, as noted by [52]. A greater dietary supply of α-tocopheryl acetate is associated with higher concentrations of this antioxidant in muscle tissue and with more efficient uptake of dietary FAs. By inhibiting in situ peroxidation of pre-existing PUFAs, α-tocopherol prevents oxidative degradation; consequently, the absolute PUFA content remains largely unchanged, while postmortem and storage-related oxidative losses are significantly reduced [53]. This dual action of vitamin E is particularly relevant for maintaining the technological, nutritional, and sensory qualities of processed products, which are more prone to free radical formation and oxidative reactions. Contrarily to vitamin E, fat addition had no effect on the proportion of fatty acids.

Like α-tocopheryl acetate supplementation, storage time significantly altered the FA profile of the rabbit hamburgers. In the F0-E200 and F2-E200 groups, the proportion of PUFAs increased (*p* < 0.05), while the share of SFAs decreased across all groups (*p* < 0.001). Storage also raised the content of long-chain n-3 FA (*p* < 0.001), thereby improving the nutritional profile by lowering the n-6/n-3 ratio (*p* < 0.001).

Demonstrating its antioxidant efficacy, dietary vitamin E significantly lowered the TBARS index in the hamburgers (*p* < 0.01; Table 9) according to other studies [54,55,56]. Contrary to expectations, dietary fat inclusion did not raise TBARS values, indicating no increase in lipid oxidation. A likely explanation is that the control animals’ hamburgers contained lipid levels comparable to those of the fat-supplemented group, thereby cancelling out any potential pro-oxidative effect of the added fat.

As expected, storage significantly elevated TBARS values in the hamburgers, confirming earlier findings [39,57,58]. The rise was especially marked in the diets without vitamin E supplementation (F0-E0 and F2-E0): on average, TBARSs increased from 0.055 mg MDA kg^−1^ in fresh patties to 0.133 mg MDA kg^−1^ after storage. Although these values remained below the sensory threshold for off-odors and off-flavors in humans [57], they still indicate appreciable oxidative change.

## 4. Conclusions

In conclusion, the dietary inclusion of 200 mg/kg of α-tocopheryl acetate significantly reduced lipid oxidation in rabbit hamburgers, both in fresh samples and after 7 days of storage. The beneficial effect of vitamin E on oxidative processes also resulted in an improvement in the dietary quality of inter- and intramuscular lipids in rabbit meat, with a tendency toward an increased proportion of PUFAs, particularly after 7 days of storage. Supra-nutritional vitamin E intake significantly improved the appearance of raw hamburgers, both at T1 and, more markedly, at T7. On cooked hamburgers, only the texture was improved, but the significant effect was limited to the T1 treatment. Dietary fat supplementation increased the content of oleic acid, reflecting the FA composition of pork fat, but no other significant effects were observed on the FAs proportion. Pork fat inclusion in the diet improved the appearance of the raw 7-day-stored hamburgers; the texture of stored and cooked hamburgers was also improved.

An increase in the concentration of n-3 FAs was observed over the storage time, leading to a decrease in the n-6/n-3 FAs ratio, with favorable implications for consumer health. Nevertheless, it is considered that the effect of storage time on the evolution of the fatty acid profile in rabbit meat warrants further and more detailed investigation.

## Figures and Tables

**Table 1:** Fatty acid profile of experimental diets (% total FAMEs).

	Experimental Diets			
	F0-E0	F0-E200	F2-E0	F2-E200
C15:0	0.50	0.48	1.35	0.49
C15:1	0.05	0.07	0.03	0.00
C16:0	18.6	18.2	20.8	21.4
C16:1	0.30	0.24	1.58	1.62
C17:0	0.17	0.15	0.37	0.35
C17:1	0.08	0.05	0.20	0.22
C18:0	2.70	2.44	7.19	7.45
C18:1 n-9	17.8	16.8	27.1	27.3
C18:2 n-6	49.6	49.4	34.3	34.1
C18:3 n-6	0.06	0.06	0.06	0.06
C18:3 n-3	8.20	9.18	5.31	5.22
C20:0	0.42	0.08	0.33	0.33
C20:1 n-9	0.62	0.14	0.57	0.60
C20:2 n-6	0.07	0.07	0.14	0.15
C21:0	0.00	0.04	0.04	0.04
C20:3 n-6	0.04	0.00	0.03	0.00
C20:3 n-3	0.00	0.00	0.12	0.12
C20:4 n-6	0.00	2.12	0.03	0.00
C22:0	0.55	0.51	0.35	0.37
C22:1 n-9	0.17	0.07	0.09	0.18
SFAs	23.0	21.9	30.4	30.3
MUFA	19.0	17.3	29.7	30.1
PUFAs	58.0	60.8	40.0	39.6
Total n-6	49.8	51.6	34.5	34.3
Total n-3	8.20	9.18	5.43	5.34
n-6/n-3	6.07	5.62	6.35	6.42

**Table 2:** Effect of supplementation of vitamin E (E200) and animal fat (F2) as well as storage time (T, days) on physico-chemical traits of raw hamburgers.

	Experimental diets (D)				Significance			RSD ^1^
	F0-E0	F0-E200	F2-E0	F2-E200	Vit E (E)	Fat (F)	ExF	D ^2^
Samples. No.	15	15	15	15				
pH								
T0	5.76	5.75	5.78	5.77	ns	ns	ns	0.14
T7	5.79	5.77	5.78	5.78	ns	ns	ns	0.07
Significance T	ns	ns	ns	ns				
RSD T	0.11	0.12	0.12	0.10				
L* value								
T0	65.3	64.7	66.2	66.5	ns	ns	ns	3.36
T7	56.8	57.4	57.4	57.6	ns	ns	ns	2.5
Significance T	<0.001	<0.001	<0.001	<0.001				
RSD T	3.0	2.4	2.9	3.4				
a* value								
T0	12.8	13.5	13.5	13.2	ns	ns	ns	1.95
T7	7.88	8.14	8.65	8.59	ns	ns	ns	1.60
Significance T	<0.001	<0.001	<0.001	<0.001				
RSD T	1.51	1.97	1.75	1.88				
b* value								
T0	9.74	10.1	10.5	10.9	ns	ns	ns	1.87
T7	6.78	6.75	7.41	6.93	ns	ns	ns	1.27
Significance T	<0.001	<0.001	<0.001	<0.001				
RSD T	1.68	1.82	1.58	1.28				
C* value								
T0	16.1	16.9	17.1	17.1	ns	ns	ns	2.5
T7	10.4	10.6	11.5	11.1	ns	ns	ns	1.7
Significance T	<0.001	<0.001	<0.001	<0.001				
RSD T	2.0	2.5	2.0	2.0				

^1^ Residual standard deviation; ^2^ diets.

**Table 3:** Effect of supplementation of vitamin E (E200) and animal fat (F2) as well as storage time (T, days) on physicochemical traits of cooked hamburgers.

	Experimental Diets (D)				Significance			RSD ^1^
	F0-E0	F0-E200	F2-E0	F2-E200	Vit E (E)	Fat (F)	ExF	D ^2^
Samples, No.	15	15	15	15				
pH								
T0	6.10	6.11	6.13	6.10	ns	ns	ns	0.12
T7	6.09	6.09	6.10	6.09	ns	ns	ns	0.08
Significance T	ns	Ns	ns	ns				
RSD T	0.09	0.11	0.10	0.09				
L* value								
T0	67.3	67.0	64.9	67.2	ns	ns	ns	4.63
T7	68.8	68.3	68.4	70.2	ns	ns	ns	4.01
Significance T	ns	Ns	<0.01	0.05				
RSD T	5.07	4.78	3.29	3.95				
a* value								
T0	5.43	5.21	5.64	5.17	ns	ns	ns	1.21
T7	4.28	4.45	4.58	4.53	ns	ns	ns	1.04
Significance T	<0.05	<0.05	<0.01	ns				
RSD T	1.20	0.99	0.99	1.28				
b* value								
T0	11.8	11.7	12.2	11.8	ns	ns	ns	0.82
T7	10.8	10.7	10.8	10.7	ns	ns	ns	1.10
Significance T	<0.01	<0.01	<0.001	<0.05				
RSD T	0.88	0.86	0.94	1.18				
C* value								
T0	13.0	12.8	13.5	12.9	ns	ns	ns	1.08
T7	11.6	11.6	11.8	11.7	ns	ns	ns	1.16
Significance T	<0.01	<0.01	<0.001	0.01				
RSD T	1.11	0.99	1.11	1.24				
Cooking loss								
T0	25.9	26.9	26.5	26.1	ns	ns	ns	3.78
T7	25.1	26.0	25.3	24.9	ns	ns	ns	1.78
Significance T	ns	Ns	ns	ns				
RSD T	3.07	3.05	2.26	3.33				

^1^ Residual standard deviation; ^2^ diets.

**Table 4:** Effect of supplementation of vitamin E (E200) and animal fat (F2) as well as storage time (T, days) on proximate composition of raw hamburgers (g/100 g).

	Experimental diets (D)				Significance			RSD ^1^
	F0-E0	F0-E200	F2-E0	F2-E200	Vit E (E)	Fat (F)	ExF	D ^2^
Samples, No.	15	15	15	15				
Moisture								
T0	67.8	68.6	67.8	68.3	ns	ns	ns	1.60
T7	67.6	68.3	67.5	67.3	ns	ns	ns	1.29
Significance T	ns	ns	ns	ns				
RSD T	1.26	1.08	1.45	1.90				
Protein								
T0	22.5	22.5	22.3	22.0	ns	ns	ns	0.82
T7	22.8	22.8	22.6	22.9	ns	ns	ns	0.58
Significance T	ns	<0.05	ns	<0.05				
RSD T	0.57	0.33	0.58	1.12				
Lipids								
T0	8.53	7.68	8.69	8.47	ns	ns	ns	1.58
T7	8.37	7.68	8.65	8.56	ns	ns	ns	1.61
Significance T	ns	ns	ns	ns				
RSD T	1.39	1.21	1.66	2.01				
Ash								
T0	1.22	1.24	1.22	1.23	ns	ns	ns	0.06
T7	1.25	1.26	1.25	1.27	ns	ns	ns	0.06
Significance T	ns	ns	ns	ns				
RSD T	0.05	0.05	0.04	0.08				

^1^ Residual standard deviation; ^2^ diets.

**Table 5:** Effect of supplementation of vitamin E (E200) and animal fat (F2) as well as storage time (T, days) on sensory attributes of hamburgers.

	Experimental Diets (D)				Significance			RSD ^1^
	F0-E0	F0-E200	F2-E0	F2-E200	Vit E (E)	Fat (F)	ExF	D ^2^
Samples, No.	15	15	15	15				
Color, raw hamburger								
T0	2.29 ^b^	2.98 ^a^	2.65 ^ab^	2.09 ^b^	ns	ns	<0.05	0.94
T7	2.24	2.71	2.66	2.38	ns	ns	ns	0.90
Significance T	ns	ns	ns	ns				
RSD T	0.98	0.87	0.72	1.06				
Appearance, raw hamburger								
T0	2.29 ^b^	2.79 ^a^	2.45 ^ab^	2.47 ^ab^	<0.05	ns	<0.05	0.44
T7	2.12	2.44	2.43	3.01	<0.05	<0.05	ns	0.78
Significance T	ns	ns	ns	<0.05				
RSD T	0.68	0.67	0.57	0.61				
Appearance, cooked hamburger								
T0	2.47	2.63	2.40	2.50	ns	ns	ns	0.90
T7	2.80	2.40	2.20	2.60	ns	ns	ns	0.87
Significance T	ns	ns	ns	ns				
RSD T	0.98	0.80	0.81	0.95				
Flavor, cooked hamburger								
T0	2.50	2.47	2.73	2.30	ns	ns	ns	0.89
T7	2.67 ^A^	1.93 ^B^	2.43 ^AB^	2.97 ^A^	ns	ns	<0.01	0.79
Significance T	ns	ns	ns	ns				
RSD T	0.80	0.86	0.75	0.94				
Texture ^3^, cooked hamburger								
T0	2.03	2.77	2.50	2.70	<0.05	ns	ns	0.86
T7	2.30	2.10	2.57	3.03	ns	<0.01	ns	0.80
Significance T	ns	<0.05	ns	ns				
RSD T	0.79	0.89	0.87	0.77				

^1^ Residual standard deviation; ^2^ diets; ^3^ texture: tenderness and juiciness; ^a,b^ Values within a row with different superscripts differ significantly at *p* < 0.05; ^A,B^ Values within a row with different superscripts differ significantly at for *p* < 0.01.

**Table 6:** Effect of supplementation of vitamin E (E200) and animal fat (F2) as well as storage time (T, days) on the saturated fatty acids proportion (% total FAMEs) of raw hamburgers.

	Experimental Diets (D)				Significance			RSD ^1^
	F0-E0	F0-E200	F2-E0	F2-E200	Vit E (E)	Fat (F)	ExF	D ^2^
Samples, No.	15	15	15	15				
<C10:0								
T0	1.02	1.22	0.91	0.95	ns	ns	ns	0.63
T7	0.37	0.63	0.47	0.71	ns	ns	ns	0.57
Significance T	<0.001	<0.05	ns	ns				
RSD T	0.43	0.63	0.62	0.69				
C10:0								
T0	0.30	0.32	0.24	0.30	ns	ns	ns	0.15
T7	0.37	0.48	0.32	0.32	ns	<0.05	ns	0.18
Significance T	ns	<0.05	ns	ns				
RSD T	0.13	0.19	0.13	0.19				
C12:0								
T0	0.24	0.29	0.19	0.28	ns	ns	ns	0.14
T7	0.39	0.34	0.31	0.33	ns	ns	ns	0.13
Significance T	<0.01	ns	<0.05	ns				
RSD T	0.12	0.16	0.12	0.13				
C14:0								
T0	3.21	3.21	3.04	3.08	ns	ns	ns	0.38
T7	4.56	3.90	4.04	3.70	ns	ns	ns	1.02
Significance T	<0.001	ns	<0.01	<0.05				
RSD T	0.53	0.93	0.78	0.79				
C15:0								
T0	0.55	0.61	0.57	0.52	ns	ns	ns	0.11
T7	0.74	0.48	0.68	0.59	<0.05	ns	ns	0.32
Significance T	<0.05	ns	ns	ns				
RSD T	0.20	0.27	0.21	0.26				
C16:0								
T0	27.8	27.6	27.3	27.2	ns	ns	ns	1.52
T7	23.3	24.3	23.9	23.5	ns	ns	ns	2.15
Significance T	<0.001	<0.001	<0.001	<0.001				
RSD T	2.06	1.89	2.15	1.20				
C17:0								
T0	0.66	0.70	0.70	0.69	ns	ns	ns	0.11
T7	0.92	0.80	0.84	0.74	ns	ns	ns	0.35
Significance T	<0.01	ns	ns	ns				
RSD T	0.20	0.32	0.24	0.27				
C18:0								
T0	7.23	7.64	8.29	8.05	ns	<0.01	ns	1.05
T7	8.01	6.96	7.75	7.40	ns	ns	ns	1.58
Significance T	ns	ns	ns	ns				
RSD T	1.09	1.48	1.27	1.48				
C20:0								
T0	0.09	0.09	0.11	0.09	ns	ns	ns	0.04
T7	0.14	0.11	0.12	0.09	ns	ns	ns	0.06
Significance T	<0.001	ns	ns	ns				
RSD T	0.04	0.06	0.06	0.06				
C21:0								
T0	0.08	0.04	0.05	0.07	ns	ns	ns	0.07
T7	0.08	0.10	0.08	0.07	ns	ns	ns	0.07
Significance T	ns	<0.05	ns	ns				
RSD T	0.06	0.08	0.06	0.06				
C22:0								
T0	0.015	0.00	0.001	0.0001	ns	ns	ns	0.025
T7	0.014	0.011	0.009	0.016	ns	ns	ns	0.014
Significance T	ns	<0.01	<0.01	<0.01				
RSD T	0.04	0.01	0.01	0.01				
C23:0								
T0	0.008	0.035	0.017	0.009	ns	ns	ns	0.044
T7	0.020	0.027	0.015	0.020	ns	ns	ns	0.037
Significance T	ns	ns	ns	ns				
RSD T	0.02	0.06	0.04	0.03				
C24:0								
T0	0.007	0.011	0.001	0.001	ns	ns	ns	0.020
T7	0.000	0.006	0.007	0.011	ns	ns	ns	0.022
Significance T	ns	ns	ns	ns				
RSD T	0.01	0.03	0.02	0.02				

^1^ Residual standard deviation; ^2^ diets.

**Table 7:** Effect of supplementation of vitamin E (E200) and animal fat (F2) as well as storage time (T, days) on the monounsaturated fatty acids proportion (% total FAMEs) of raw hamburgers.

	Experimental Diets (D)				Significance			RSD ^1^
	F0-E0	F0-E200	F2-E0	F2-E200	Vit E (E)	Fat (F)	ExF	D ^2^
Samples, No.	15	15	15	15				
C14:1								
T0	0.49	0.49	0.38	0.45	ns	ns	ns	0.21
T7	0.84	0.63	0.58	0.62	ns	ns	ns	0.35
Significance T	<0.05	ns	<0.05	<0.05				
RSD T	0.43	0.24	0.20	0.22				
C15:1								
T0	0.09	0.06	0.02	0.04	ns	ns	ns	0.17
T7	0.07	0.22	0.05	0.09	ns	ns	ns	0.27
Significance T	ns	ns	<0.05	ns				
RSD T	0.22	0.37	0.04	0.13				
C16:1								
T0	7.36	6.82	5.97	6.45	ns	<0.05	ns	1.69
T7	9.46	8.27	7.90	7.96	ns	<0.05	ns	1.74
Significance T	<0.01	<0.05	<0.001	<0.05				
RSD T	1.92	1.60	1.40	1.89				
C17:1								
T0	0.27 ^BC^	0.39 ^A^	0.35 ^AB^	0.22 ^C^	ns	ns	<0.001	0.11
T7	0.58	0.45	0.42	0.47	ns	ns	ns	0.22
Significance T	<0.001	ns	ns	<0.001				
RSD T	0.15	0.19	0.19	0.16				
C18:1 n-9								
T0	25.2	24.1	26.4	26.1	<0.05	<0.001	ns	1.08
T7	23.6	23.7	25.4	25.3	ns	<0.001	ns	1.45
Significance T	<0.001	ns	<0.01	ns				
RSD T	1.17	1.32	1.04	1.53				
C20:1 n-9								
T0	0.33	0.28	0.34	0.27	<0.05	ns	ns	0.10
T7	0.54	0.37	0.42	0.36	ns	ns	ns	0.25
Significance T	<0.01	ns	ns	ns				
RSD T	0.18	0.19	0.21	0.20				

^1^ Residual standard deviation; ^2^ diets. ^A–C^ Values within a row with different superscripts differ significantly at for *p* < 0.01.

**Table 8:** Effect of supplementation of vitamin E (E200) and animal fat (F2) as well as storage time (T, days) on the polyunsaturated fatty acids proportion (% total FAMEs) of raw hamburgers.

	Experimental Diets (D)				Significance			RSD ^1^
	F0-E0	F0-E200	F2-E0	F2-E200	Vit E (E)	Fat (F)	ExF	D ^2^
Samples, No.	15	15	15	15				
C18:2 n-6								
T0	20.2	21.2	20.5	20.9	ns	ns	ns	1.8
T7	20.3	22.2	21.2	22.1	ns	ns	ns	2.7
Significance T	ns	ns	ns	ns				
RSD T	1.71	2.28	2.40	2.79				
C18:3 n-6								
T0	0.08	0.08	0.07	0.05	ns	ns	ns	0.03
T7	0.11	0.11	0.09	0.11	ns	ns	ns	0.07
Significance T	ns	ns	ns	*p* < 0.05				
RSD T	0.06	0.06	0.04	0.06				
C18:3 n-3								
T0	3.13	3.27	3.02	3.06	ns	ns	ns	0.30
T7	4.58	4.68	4.34	4.29	ns	ns	ns	0.60
Significance T	<0.001	<0.001	<0.001	<0.001				
RSD T	0.45	0.54	0.50	0.39				
C20:2 n-6								
T0	0.19	0.20	0.17	0.17	ns	ns	ns	0.12
T7	0.24	0.18	0.24	0.16	ns	ns	ns	0.11
Significance T	ns	ns	<0.05	ns				
RSD T	0.15	0.11	0.09	0.09				
C20:3 n-6								
T0	0.28	0.30	0.36	0.05	ns	ns	ns	0.40
T7	0.05	0.01	0.03	0.03	ns	ns	ns	0.04
Significance T	<0.05	ns	<0.01	ns				
RSD T	0.25	0.39	0.31	0.06				
C20:3 n-3								
T0	0.56	0.70	0.58	0.76	ns	ns	ns	0.41
T7	0.56	0.55	0.55	0.57	ns	ns	ns	0.22
Significance T	ns	ns	ns	ns				
RSD T	0.35	0.26	0.41	0.27				
C20:4 n-6								
T0	0.03	0.05	0.03	0.01	ns	ns	ns	0.05
T7	0.06	0.07	0.06	0.07	ns	ns	ns	0.05
Significance T	ns	ns	<0.05	<0.01				
RSD T	0.04	0.06	0.03	0.05				
C20:5 n-3								
T0	0.01	0.02	0.01	0.00	ns	ns	ns	0.03
T7	0.02	0.01	0.02	0.01	ns	ns	ns	0.01
Significance T	ns	ns	ns	<0.01				
RSD T	0.02	0.03	0.02	0.01				
C22:2 n-6								
T0	0.55	0.32	0.38	0.27	ns	ns	ns	0.49
T7	0.12	0.33	0.12	0.24	ns	ns	ns	0.28
Significance T	ns	ns	<0.001	ns				
RSD T	0.59	0.47	0.15	0.21				
C22:6 n-3								
T0	0.012	0.001	0.020	0.002	ns	ns	ns	0.045
T7	0.000	0.009	0.000	0.021	ns	ns	ns	0.032
Significance T	ns	ns	ns	ns				
RSD T	0.031	0.022	0.055	0.041				

^1^ Residual standard deviation; ^2^ diets.

**Table 9:** Effect of supplementation of vitamin E (E200) and animal fat (F2) as well as storage time (T, days) on fatty acid (FA) classes (% total FAMEs) and TBARS value (mg MDA/kg) of raw hamburgers.

	Experimental Diets (D)				Significance			RSD ^1^
	F0-E0	F0-E200	F2-E0	F2-E200	Vit E (E)	Fat (F)	ExF	D ^2^
Samples, No.	15	15	15	15				
Saturated Fatty Acids (SFAs)								
T0	41.2	41.7	41.4	41.2	ns	ns	ns	1.53
T7	38.9	38.2	38.6	37.5	ns	ns	ns	2.54
Significance T	<0.01	<0.001	<0.001	<0.001				
RSD T	2.02	1.98	1.80	2.53				
Monounsaturated Fatty Acids (MUFAs)								
T0	33.8	32.2	33.5	33.6	ns	ns	ns	2.37
T7	35.1	33.7	34.8	34.9	ns	ns	ns	2.48
Significance T	Ns	ns	ns	ns				
RSD T	2.21	2.08	2.03	3.20				
Polyunsaturated Fatty Acids (PUFAs)								
T0	25.0	26.1	25.1	25.3	ns	ns	ns	2.09
T7	26.0	28.2	26.7	27.6	<0.05	ns	ns	2.86
Significance T	ns	<0.05	ns	<0.05				
RSD T	1.81	2.38	2.62	3.05				
Total n-6 FA								
T0	21.3	22.1	21.5	21.4	ns	ns	ns	1.88
T7	20.9	22.9	21.8	22.7	<0.05	ns	ns	2.72
Significance T	ns	ns	ns	ns				
RSD T	1.55	2.50	2.38	2.76				
Total n-3 FA								
T0	3.70	3.98	3.62	3.82	ns	ns	ns	0.47
T7	5.15	5.25	4.91	4.89	ns	ns	ns	0.61
Significance T	<0.001	<0.001	<0.001	<0.001				
RSD T	0.53	0.53	0.61	0.51				
n-6/n-3 ratio								
T0	5.84	5.61	6.01	5.64	ns	ns	ns	0.77
T7	4.09	4.44	4.53	4.66	ns	ns	ns	0.73
Significance T	<0.001	<0.01	<0.001	<0.001				
RSD T	0.59	0.88	0.88	0.58				
TBARSs								
T0	0.059 ^a^	0.034 ^b^	0.051 ^a^	0.047 ^ab^	<0.01	ns	<0.05	0.016
T7	0.132	0.083	0.133	0.058	<0.01	ns	ns	0.076
Significance T	<0.001	<0.001	<0.05	<0.05				
RSD T	0.043	0.026	0.095	0.013				

^1^ Residual standard deviation; ^2^ diets. ^a,b^ Values within a row with different superscripts differ significantly at *p* < 0.05.

## Data Availability

The datasets analyzed in the present study are available from the corresponding author upon request.

## Supplementary Materials

The following supporting information can be downloaded at: https://www.mdpi.com/article/10.3390/ani15121804/s1, Table S1: Formulation (g/kg) of the experimental diets; Table S2: Chemical composition (g/kg as fed), α-tocopheryl acetate (mg/kg as feed) and energy content (MJ/kg as fed) of the experimental diets.
