# Supplementary material for: Influence of Dietary Alpha-Tocopheryl Acetate (Vitamin E) and Animal Fat on the Chemical Composition, Fatty Acid Profile, Lipid Stability and Sensory Traits of Fresh and Stored Hamburgers from Rabbit Meat
Source: Animals (Basel). 2025 Jun 19;15(12):1804. doi: 10.3390/ani15121804 (PMC12189813; doi:10.3390/ani15121804)
Supplement: Supplementary file 1 [file animals-15-01804-s001.zip › animals-3656105-supplementary.pdf]

Supplementary Materials

Influence of Dietary Alpha-Tocopheryl Acetate (Vitamin E) and Animal Fat on the Chemical Composition, Fatty Acid Profile, Lipid Stability and Sensory Traits of Fresh and Stored Hamburgers from Rabbit Meat

Bianca Palumbo [1], **Maria Elena** Cossu [2] **and Antonella** Dalle Zotte [1,*]

[1] Department of Animal Medicine, Production and Health, Agripolis, University of Padova, 35122 Padova, Italy; biancafederica.palumbo@phd.unipd.it
[2] Department of Animal Production, Faculty of Agronomy, University of Buenos Aires, Buenos Aires C1053ABH, Argentina; mcossu@agro.uba.ar
* Correspondence: antonella.dallezotte@unipd.it

Table S1. Formulation (g/kg) of the experimental diets

	Experimental diets	
	**F0-E0**	**F2-E0**
Alfalfa hay 17% CP	300	300
Barley	240	200
Wheat bran	260	260
Sugar beet pulp	75.0	75.0
Soybean meal 44% CP	45.0	45.0
Sunflower meal 30% CP	45.0	65.0
Pork lard	0.00	20.0
Cane molasses	23.0	23.0
Dicalcium phosphate	3.50	3.50
Salt	3.50	3.50
Vitamin-mineral premix	3.00	3.00
DL-methionine	1.00	1.00
Coccidiostatic	1.00	1.00

CP= crude protein; F0-E0= no fat, no vitamin E; F2-E0= 2 % fat, no vitamin E

Table S2. Chemical composition (g/kg as fed), α-tocopheryl acetate (mg/kg as feed) and and energy content (MJ/kg as fed) of the experimental diets

	**Experimental diets**			
	**F0-E0**	**F0-E200**	**F2-E0**	**F2-E200**
Dry matter	899	899	899	902
Crude protein (CP)	147	151	153	155
Ether extract (EE)	26.1	25.2	43.2	45.1
Crude fiber (CF)	138	139	142	136
Ash	71.0	71.9	71.9	72.2
Neutral-detergent fibre (NDF)	342	343	344	338
Acid-detergent fibre (ADF)	160	173	168	169
Acid-detergent lignine (ADL)	29.7	36.8	38.7	37.0
α-tocopheryl acetate	55.7	200	66.5	198
Gross energy (GE)	16.3	16.3	16.7	16.9

F0-E0 = no fat, no vitamin E; F0-E200 = no fat, 200 mg/kg of α-tocopheryl acetate; F2-E0 = 2% fat, no vitamin E; F2-E200 = 2% fat, 200 mg/kg of α-tocopheryl acetate
